# Human Metapneumovirus: Insights from a Ten-Year Molecular and Epidemiological Analysis in Germany

**DOI:** 10.1371/journal.pone.0088342

**Published:** 2014-02-05

**Authors:** Janine Reiche, Sonja Jacobsen, Katrin Neubauer, Susi Hafemann, Andreas Nitsche, Jeanette Milde, Thorsten Wolff, Brunhilde Schweiger

**Affiliations:** 1 Division of Influenza Viruses and Other Respiratory Viruses, National Reference Centre for Influenza, Robert Koch Institute, Berlin, Germany; 2 Centre for Biological Threats and Special Pathogens, Highly Pathogenic Viruses, Robert Koch Institute, Berlin, Germany; Kliniken der Stadt Köln gGmbH, Germany

## Abstract

Human metapneumovirus (HMPV) is a cause of respiratory tract illness at all ages. In this study the epidemiological and molecular diversity among patients of different ages was investigated. Between 2000–2001 and 2009–2010, HMPV was detected in 3% (138/4,549) of samples from outpatients with influenza-like illness with a new, sensitive real-time RT-PCR assay. Several hundred (797) clinical specimens from hospitalized children below the age of 4 years with acute respiratory illness were investigated and HMPV was detected in 11.9% of them. Investigation of outpatients revealed that HMPV infections occurred in individuals of all ages but were most prevalent in children (0–4 years) and the elderly (>60 years). The most present clinical features of HMPV infections were cough, bronchitis, fever/shivers and pneumonia. About two thirds of HMPV-positive samples were detected in February and March throughout the study period. Molecular characterization of HMPV revealed a complex cyclic pattern of group dominance where HMPV subgroup A and B viruses predominated in general for three consecutive seasons. German HMPV represented all genetic lineages including A1, A2, B1, B2, sub-clusters A2a and A2b. For Germany, not only time-dependent circulation of lineages and sub-clusters was observed but also co-circulation of two or three predominant lineages. Two newly emerging amino acid substitutions (positions 223 and 280) of lineage B2 were detected in seven German HMPV sequences. Our study gives new insights into the molecular epidemiology of HMPV in in- and outpatients over a time period of 10 years for the first time. It is one of only few long-term surveillance studies in Europe, and allows comparative molecular analyses of HMPV circulating worldwide.

## Introduction

Human metapneumovirus (HMPV) is a recently detected paramyxovirus that has been linked to acute respiratory illness in individuals of all ages [1]. Almost all children by 5 years of age had been infected with HMPV [1], [2]. In children, HMPV symptomatology is similar to RSV whereas in adults and elderly subjects HMPV usually causes influenza-like illness (ILI) and colds [3], [4]. More severe courses of disease were preferentially observed in young children, elderly individuals and immunocompromised hosts [5].

Genotyping studies have classified circulating HMPV into the two subgroups A and B [1], [6] in which the main differences are found in the SH and G protein genes [6]. Among HMPV genes a high level of sequence conservation was found for the F gene that is assumed to be the principal target for cross-lineage neutralizing antibody response [6], [7]. Within each group the nucleotide and amino acid identity of the F gene was 94.3–100% and 98.3–100%, respectively [8]. Between subgroup A and B the identity was 83.0–83.6% and 94.1–95.4% for the F gene nucleotides and derived amino acid sequences, respectively [8]. The HMPV F gene codes for a fusion protein of 539 amino acids in length, harbouring 14 conserved cysteine residues and 3 N-glycosylation sites [9], [10]. Based on phylogenetic analyses, HMPV strains can be further classified into four genetic lineages, named A1, A2, B1 and B2 [11], [12] as well as into the previously assigned sub-clusters A2a and A2b [13]. Intergenotypic comparison of the amino acid sequence of the F gene identified a number of conserved amino acid residues specific for each group or lineage [9], [11], [14].

Since its initial description HMPV has been identified in all five continents. In temperate climates the highest number of HMPV infections is diagnosed at the end of winter or in early spring [15]–[19], whereas in the subtropics it peaks in spring and summer [20].

Despite the worldwide investigation and detection of HMPV there is limited information about frequency and lineage distribution in multiple consecutive seasons. This study describes for the first time the prevalence, the genetic variability and the circulation pattern of HMPV over a ten year period in Germany.

## Materials and Methods

### Ethics Statement

Specimens were taken from patients with influenza-like illness (ILI) who gave verbal consent for laboratory examination without documentation. Samples were sent by sentinel practitioners to the National Reference Centre for Influenza at the Robert Koch Institute, Germany for acute respiratory infections (ARI) surveillance purposes in Germany. Specimens from hospitalized children with ARI were collected by the attending physicians in hospitals. The analyses of all data were done anonymously. Ethic committee approval was not required since such a sentinel surveillance is covered by German legislation (§13, §14, Protection against Infection Act).

### Clinical Samples

Two panels of specimen were tested in this study. Specimens from patients with influenza-like illness (ILI) were collected by the German National Reference Centre for Influenza at the Robert Koch Institute, mainly provided by about 150 medical practices participating in the Influenza Surveillance Scheme (National German Working Group Influenza). The Influenza-like illness case definition is a sudden onset of symptoms with at least one of the following systemic symptoms: fever or feverishness, malaise, headache, myalgia and at least one of the following respiratory symptoms: cough, sore throat, shortness of breath and corresponds to the definition of the European Centre for Disease Prevention and Control (ECDC). The amount of analysed specimens was 15% of the sample receipt per calendar week in each age group. All samples were tested negative for influenza virus and RSV by RT-PCR. Specimens were retrospectively investigated for HMPV from calendar weeks 41 to 18 (October to the end of April) from the seasons 2000–2001 to 2009–2010. To describe the circulation of HMPV in the German population, specimens were analysed from five different age groups: 0–4 years, 5–15 years, 16–34 years, 35–60 years, >60 years. The second test panel were specimens from hospitalized children with acute respiratory illness (ARI). ARI was defined by the National German Working Group Influenza at the Robert Koch Institute as patients with Bronchitis or Pneumonia or acute Pharyngitis with or without fever. From 2000–2001 to 2009–2010 two hospitals sent specimens from patients aged between 0 and 4 years to the National Reference Centre for Influenza. All specimens were initially tested for influenza virus and RSV. Only influenza virus- and RSV-negative samples available from calendar weeks 41 to 18 were investigated for HMPV. Symptoms and diagnoses for outpatients and inpatients were requested from medical practices and hospitals over the whole period; bronchitis was requested for outpatients (ILI patients) in only three out of ten seasons (2000–2001, 2008–2009 and 2009–2010) due to changes in the clinical query.

Specimens collected included human nasopharyngeal aspirates and nasal or throat swabs. Immediately upon arrival swabs were resuspended or adjusted to 3 ml of sterile minimal essential medium with HEPES (Gibco BRL, Eggenstein, Germany) and 100 U/ml PEN/STR (Gibco BRL), aliquoted and stored at −70°C. Nasopharyngeal aspirates were adjusted to a volume of 3 ml using the medium described before.

### PCR

RNA was extracted from 300 µl culture supernatant using the MagAttract Viral RNA M48 Kit (Qiagen, Hilden, Germany) and eluted in 80 µl elution buffer. Alternatively, RNA was extracted using the MagNA Pure 96 DNA and Viral NA Small Volume Kit (Roche Deutschland Holding GmbH, Mannheim, Germany) from 200 µl culture supernatant with an elution volume of 50 µl and using the RTP DNA/RNA Virus Mini Kit (Invitek, Berlin, Germany) from 400 µl culture supernatant with an elution volume of 60 µl, respectively. 25 µl of extracted RNA were subjected to cDNA synthesis applying 200 U M-MLV Reverse Transcriptase (Invitrogen, Karlsruhe, Germany) in a total volume of 40 µl.

Screening of respiratory samples for HMPV was performed with a new validated, specific and sensitive two-step real-time RT-PCR assay described below. To allow for a group-independent detection, their genetically most conserved region was chosen for PCR amplification. The specificity of the assays was evaluated with nucleic acids of various circulating HMPV lineages (A1, A2, B1 and B2) and other respiratory pathogens including influenza virus types A and B, human parainfluenzavirus 1–4, human respiratory syncytial virus A and B, human adenovirus types 2–4, human echovirus 6, 9, 11 and 19, human coxsackievirus A4 and B3, human rhinovirus 1B and 37, *Streptococcus pneumoniae* type 14 and 23, *Staphylococcus aureus* type 4 and 5 and *Chlamydia pneumoniae*. All of the nucleic acids had been previously tested positive with specific PCR assays. The HMPV assay showed 100% specificity since no other viral or bacterial respiratory pathogen was detected.

PCR efficiency was determined with the help of plasmids containing the respective PCR target sequence. The assay displayed a dynamic range from 10^6^ to 10^1^ target copies with a high standard curve correlation of R^2^ = 0.987. For determination of the reproducibility intra- and interassay variability was done using plasmids. The range of the threshold cycle (C_T_) values varied between 0.09 and 0.22 C_T_ for the intraassay and the interassay variation was between 0.41 and 0.66 C_T_. The 95% detection probability (Probit analysis) was found to be 10.1 genome equivalents per reaction.

Clinical samples were analyzed by our new developed real-time PCR on the ABI PRISM® 7500 Sequence Detection System (Applied Biosystems, Weiterstadt, Germany) or LightCycler® 480 Real-Time PCR System (Roche Deutschland Holding GmbH), respectively in a total reaction volume of 25 µl. The reaction contained 1×PCR buffer (200 mM Tris-HCl [pH 8.4], 500 mM KCl), 5 mM MgCl_2_ (Invitrogen), 0.1 mM dNTP (Invitrogen) with dUTP (GE Healthcare, Munic, Germany), 0.5 U Platinum *Taq* DNA polymerase (Invitrogen), primers (Metabion, Martinsried, Germany): HMPV F S (gCTCCgTAATYTACATggTgCA); HMPV F S1 (gAAgCTCYgTgATTTACATggTYCA); HMPV F AS (gACCCTgCARTCTgACAATACCA); HMPV F AS1 (AgTKgATCCTgCATTTTTACAATACCA) and probes: (Applied Biosystems, Foster City, United States and Tib Molbiol, Berlin, Germany) HMPV F TMGB (F-CCYTgCTggATAgTAAAA-MGB); HMPV F TMGB1 (F-CCTTgTTggATAATCAA-MGB) in differing concentrations, and 3 µl of cDNA. Amplification was carried out at 95°C for 5 min, followed by 45 cycles at 95°C for 15 s and 60°C for 30 s.

### PCR for Molecular Analysis of the F Protein Gene and Sequencing

A number of 95 of HMPV-positive samples were selected for partial amplification of the F protein gene by either external or semi-nested PCR. The first amplification was performed with 5 µl of cDNA in a 50 µl reaction by using 250 nM of each of the primers (Metabion) HMPV-3637-F (gTYAgCTTCAgTCAATTCAACAgAAg) [13], HMPV-4192-R1 (CAgTgCAACCATACTgATRggATg) and HMPV-4192-R2 (TAgTgCAACCATACTgATRgggTg), 100 µM deoxynucleoside triphosphates, 3 mM MgCl_2_, 1 U Platinum *Taq* DNA polymerase (Invitrogen) and PCR buffer (200 mM Tris-HCl [pH 8.4], 500 mM KCl). Amplification was carried out at 94°C for 5 min, followed by 40 cycles at 94°C for 30 s, 60°C for 30 s, and 72°C for 45 s, with a final extension at 72°C for 10 min. The amplified products of 555 bp were analyzed by electrophoresis on a 1.5% agarose gel. In the case of negative results, 5 µl of the external PCR reaction were used for semi-nested PCR which was performed in a 50 µl reaction with 250 nM each of the primer HMPV-3637-F and HMPV-4164-R (CCTgTgCTRACTTTgCATggg), respectively. The cycling protocol was the same as for the external PCR except for the annealing temperature which was 66°C. The nested amplicons of 527 bp were visualized by agarose gel electrophoresis as well. The PCR products were purified either directly with QIAquick PCR Purification Kit (Qiagen, Hilden, Germany) or from agarose gels with JETquick spin column technique (Genomed, Löhne, Germany) according to the manufacturer’s instructions. Purified PCR products were cycle sequenced in the forward and the reverse directions with primer pairs previously used for external and semi-nested PCR, respectively, in a 3130×l Genetic Analyzer (Applied Biosystems) by using the BigDye Terminator v3.1 Cycle Sequencing Kit (Applied Biosystems).

### Phylogenetic Analysis

A multiple sequence alignment was compiled from a part (473 nt) of the FI subunit of the F gene using ClustalW in the Bioedit version 7.0.9 [21]. Maximum likelihood tree was constructed by using Geneious 5.0.4 [22]. The reliability of the branching order was estimated by performing 1,000 bootstrap replicates with the HKY85 model. Phylogenetic analysis included representative sequences of the described HMPV lineages and subgroups. GenBank accession numbers and the country of isolation of the reference sequences are given in the supporting information (Table S1). The tree was manually edited in Corel Draw 12 program.

Pairwise nucleotide and amino acid distances within and between groups were calculated using MEGA and determined distances described in terms of mean and standard deviation.

### Nucleotide Sequence Accession Numbers

The GenBank accession numbers of the nucleotide sequences obtained in the present study are HQ456546 to HQ456640.

### Statistical Analysis

The SPSS software version 17.0 was used for statistical analysis of data. Differences between mild and intensive HMPV seasons were statistically analyzed using the chi-squared test and frequency of HMPV-positive inpatients and outpatients using the unpaired t-test. Probit analysis was undertaken to determine the assay’s limit of detection (95% confidence interval, CI). Odds ratio (OR) and CI were calculated according to diagnosis and symptoms. OR was calculated as the ratio of the odds of a diagnosis/symptom in HMPV-positive patients to the odds of it in HMPV-negative patients. HMPV-negative patients belonged to the study population and were therefore symptomatic patients with ILI or ARI without a detectable HMPV infection.

## Results

### Frequency and Distribution of HMPV

During the ten-year study period from seasons 2000–2001 to 2009–2010 more than 4,500 clinical specimens were tested by a newly developed and validated sensitive and specific HMPV in-house real-time RT-PCR assay. For generation of population based data, a panel of specimens from patients with ILI was analysed. Sixty-six (48%) of the ILI specimens were from females and the median age of HMPV-positive specimens was 7 years (range: 0–88 years). Specimens investigated herein were obtained from patients of five different age groups (0–4 years, 5–15 years, 16–34 years, 35–60 years, and >60 years) to elucidate the occurrence of HMPV in the German population. On average 3% (138) were found to be positive (Table 1). HMPV infections were detected in patients from all age groups but were most common among children between 0 and 4 years (6.9%) and individuals above 60 years (4.6%). Whereas in children between 0 and 4 years HPMV was found in every season with the lowest positive rate of 2.2% and the highest rate of 16.8%, in adults >60 years the rate of positive samples peaked in every other season being either low (0%–2%) or high (7.5%–14.2%) (Table 1). The virus was detected in every season. Season 2003–2004 (6.8%) and season 2009–2010 (4.7%) were characterized by a very intensive circulation of HMPV in Germany (estimated using chi-square test, *P<*0.001) (Table 1). As approximately two thirds of positive HMPV samples were detected between calendar weeks 3 and 16, we concluded that HMPV peak activity occurred mainly in February and March.

**Table 1:** Frequency of HMPV, by age group and epidemic season.

	Frequency of HMPV in % (positive/analyzed sample)					
Epidemic season (yr)	Total	0–4 years	5–15 years	16–34 years	35–60 years	>60 years
2000–2001	1.2 (4/330)	3.1 (3/97)	2.0 (1/49)	0 (0/63)	0 (0/72)	0 (0/49)
2001–2002	3.5 (15/429)	2.2 (1/44)	4.1 (5/120)	2.0 (2/100)	2.0 (2/99)	7.5 (5/66)
2002–2003	2.5 (11/440)	8.0 (6/75)	1.8 (2/109)	1.5 (2/127)	1.0 (1/94)	0 (0/35)
2003–2004	**6.8 (25/365)**	12.0 (9/75)	**6.2 (6/97)**	1.5 (1/63)	**4.4 (3/67)**	9.5 (6/63)
2004–2005	1.2 (10/803)	3.0 (4/133)	0.4 (1/260)	1.3 (2/149)	0.5 (1/171)	2.2 (2/90)
2005–2006	3.6 (12/333)	3.5 (2/57)	0.7 (1/126)	**5.6 (3/54)**	1.6 (1/61)	**14.2 (5/35)**
2006–2007	2.5 (11/434)	7.4 (10/136)	0 (0/127)	0 (0/72)	1.8 (1/57)	0 (0/42)
2007–2008	3.8 (16/411)	10 (6/60)	3.0 (4/132)	1.4 (1/68)	1.9 (2/103)	6.3 (3/48)
2008–2009	2.2 (12/540)	5.4 (5/93)	2.0 (3/149)	0.9 (1/108)	2.3 (3/130)	0 (0/60)
2009–2010	4.7 (22/464)	**16.8 (13/77)**	3.0 (5/164)	0 (0/94)	1.1 (1/93)	8.3 (3/36)
Total	3.0 (138/4549)	6.9 (59/847)	2.1 (28/1333)	1.3 (12/898)	1.6 (15/947)	4.6 (24/524)

### Frequency of HMPV in Hospitalized Paediatric Patients Compared to Outpatients

To study the frequency of HMPV and its impact in children under the age of 4 years a panel of 797 clinical specimens from hospitalized children (<4 year) with ARI were collected from seasons 2000–2001 to 2009–2010. PCR assay revealed the presence of HMPV in 11.9% (95) of the inpatient samples (Table 2). Frequency of HMPV was in the range of 0% and 32.8% with an intensive circulation of HMPV in the seasons 2001–2002 (32.3%) and 2009–2010 (32.8%) (estimated using chi-square test, *P* = 0.001). Whereas in the seasons 2000–2001 to 2005–2006 the proportion of positive samples tended to increase and decrease from one to the next season, the rate remained relatively high from season 2005–2006 to 2009–2010.

**Table 2:** Frequency of HMPV in 0 to 4 years old inpatients and outpatients.

	Inpatients			Outpatients		
Epidemic season (yr)	No. of samples analyzed	No. of positive samples	Prevalence (%)	No. of samples analyzed	No. of positive samples	Prevalence (%)
2000–2001	25	0	0	97	3	3.1
2001–2002	34	11	32.3	44	1	2.2
2002–2003	71	1	1.4	75	6	8.0
2003–2004	117	17	14.5	75	9	12.0
2004–2005	140	7	5.0	133	4	3.0
2005–2006	97	17	17.5	57	2	3.5
2006–2007	95	8	8.4	136	10	7.4
2007–2008	90	6	6.7	60	6	10
2008–2009	64	7	10.9	93	5	5.4
2009–2010	64	21	32.8	77	13	16.8
Total	797	95	11.9	847	59	6.9

In comparison to outpatients of the same age, frequency of HMPV in inpatients (11.9%) was about twice as high as in outpatients (6.9%). The proportion of HMPV tended to be higher in inpatients, whereas significant differences were only determined for the seasons 2001–2002 (*P*≤0.001), 2005–2006 (*P*≤0.01) and 2009–2010 (P<0.5) (Table 2).

### Clinical Diagnosis Associated with HMPV

The most frequent symptoms among all ILI cases positive for HMPV were cough (94%), sudden onset (92%), fever/shivers (88%) and muscle pain/headache (72%) (Table 3). Bronchitis (17%), fever (17%), or pneumonia (7%) were seen less frequently. The ORs for diagnoses and symptoms associated with positivity for HMPV are depicted in Table 4. HMPV infections were associated with cough (OR 4.54; 95% CI = 2.22 to 9.31), fever/shivers (OR 2.01; 95% CI = 1.21 to 3.36), bronchitis (OR 2.76; 95% CI = 1.43 to 5.34), and pneumonia (OR 3.39; 95% CI = 1.67 to 6.87). Despite the general association of HMPV with these symptoms and diagnoses there were differences with regard to the age. Cough (OR 9.96; 95% CI = 2.41 to 41.15) and bronchitis (OR 2.70; 95% CI = 1.09 to 6.66) were associated with HMPV in patients younger than 4 years. Fever/shivers (OR 4.29; 95% CI = 1.26 to 14.58) in patients older than 60 years (Table 4).

**Table 3:** Comparison of the clinical features among patients with HMPV (outpatients with ILI) in different age groups.

Variables	Number of positive samples (%)					
Age group	total (n = 138)	0–4 years (n = 59)	5–15 years (n = 28)	16–34 years (n = 12)	35–60 years (n = 15)	>60 years (n = 24)
Age (median)	7	2	7	31	47	67
Females	66	23	13	8	7	15
Diagnosis/Symptom						
Cough	130 (94)	54 (96)	26 (93)	12 (100)	13 (87)	22 (92)
Sudden onset	127 (92)	51 (91)	25 (89)	12 (100)	15 (100)	23 (96)
Fever/Shivers	121 (88)	49 (88)	27 (96)	8 (67)	13 (87)	21 (88)
Muscle pain/Headache	100 (72)	28 (50)	25 (89)	11 (92)	14 (93)	21 (88)
Bronchitis^a^	17 (46)	10 (48)	3 (38)	0	1 (25)	3 (100)
Fever	23 (17)	9 (16)	6 (21)	0	1 (7)	7 (29)
Pneumonia	9 (7)	3 (5)	2 (7)	0	1 (7)	3 (13)

a Bronchitis was requested in three out of ten seasons (number of HMPV-positive samples correlated with bronchitis n = 37, within age groups: 0–4 years n = 21; 5–15 years n = 8; 16–34 years n = 1; 35–60 years n = 4; >60 years n = 3).

**Table 4:** Odds ratio of HMPV-positive patients with influenza-like illness (outpatients) according to diagnoses and symptoms.

	total		0–4 years		5–15 years		16–34 years		35–60 years		>60 years	
Diagnosis/Symptom	OR	95% CI	OR	95% CI	OR	95% CI	OR	95% CI	OR	95% CI	OR	95% CI
Cough	4.54	2.22, 9.31	9.96	2.41, 41.15	3.55	0.84, 15.05	−	−	1.81	0.41, 8.09	2.85	0.66, 12.33
Sudden onset	1.13	0.61, 2.11	1.52	0.68, 3.42	0.47	0.14, 1.60	−	−	−	−	3.32	0.44, 24.98
Fever/Shivers	2.01	1.21, 3.36	2.26	1.01, 5.07	5.03	0.68, 37.24	0.61	0.18, 2.03	1.96	0.43, 8.65	4.29	1.26, 14.58
Muscle pain/Headache	0.80	0.55, 1.17	1.19	0.70, 2.01	1.77	0.53, 5.92	1.71	0.22, 13.34	2.64	0.34, 20.25	2.11	0.62, 7.22
Bronchitis^a^	2.76	1.43, 5.34	2.70	1.09, 6.66	3.32	0.77, 14.31	0	−	0.94	0.10, 9.20	0	−
Fever	1.04	0.66, 1.64	0.74	0.36, 1.54	1.70	0.68, 4.26	0	−	0.36	0.05, 2.72	2.33	0.94, 5.82
Pneumonia	3.39	1.67, 6.87	1.51	0.44, 5.13	5.83	1.28, 26.53	0	−	3.10	0.39, 24.66	3.62	0.99, 13.19

OR: odds ratio; CI: confidence interval,

a Bronchitis was requested in three out of ten seasons.

The comparison of children (0–4 years) with HMPV under hospitalized or ambulant conditions showed that cough (OR 2.59; 95% CI = 1.53 to 4.38) is associated with HMPV in inpatients whereas in outpatients cough (OR 9.96; 95% CI = 2.41 to 41.15), fever/shivers (OR 2.26; 95% CI = 1.01 to 5.07) and bronchitis (OR 2.70; 95% CI = 1.09 to 6.66) were associated with HMPV (Table 5).

**Table 5:** Comparison of odds ratio from HMPV-positive inpatients and outpatients (children 0–4 years old) according to diagnoses and symptoms.

	Inpatients (n = 797)			Outpatients (n = 847)		
	No. of positivesamples (%) (n = 95)	OR	95% CI	No. of positivesamples (%) (n = 59)	OR	95% CI
Age/Years (median)	<1			2		
Females	33			23		
Diagnosis/Symptom						
Cough	76 (80)	2.59	1.53, 4.38	54 (96)	9.96	2.41, 41.15
Sudden onset	29 (31)	0.93	0.59, 1.48	51 (91)	1.52	0.68, 3.42
Fever/Shivers	24 (25)	1.28	0.78, 2.10	49 (88)	2.26	1.01, 5.07
Muscle pain/Headache	1 (1)	0.56	0.07, 4.36	28 (50)	1.19	0.70, 2.01
Bronchitis^a^	42 (44)	1.45	0.94, 2.24	10 (48)	2.70	1.09, 6.66
Fever	21 (22)	1.27	0.76, 2.14	9 (16)	0.74	0.36, 1.54
Pneumonia	30 (32)	1.49	0.93, 2.38	3 (5)	1.51	0.44, 5.13

OR: odds ratio; CI: confidence interval,

a Bronchitis in outpatients was requested in three out of ten seasons (number of HMPV-positive samples n = 21, number of HMPV-positive specimens with bronchitis n = 10).

### Genetic Diversity of HMPV in Germany

**Figure 1:**
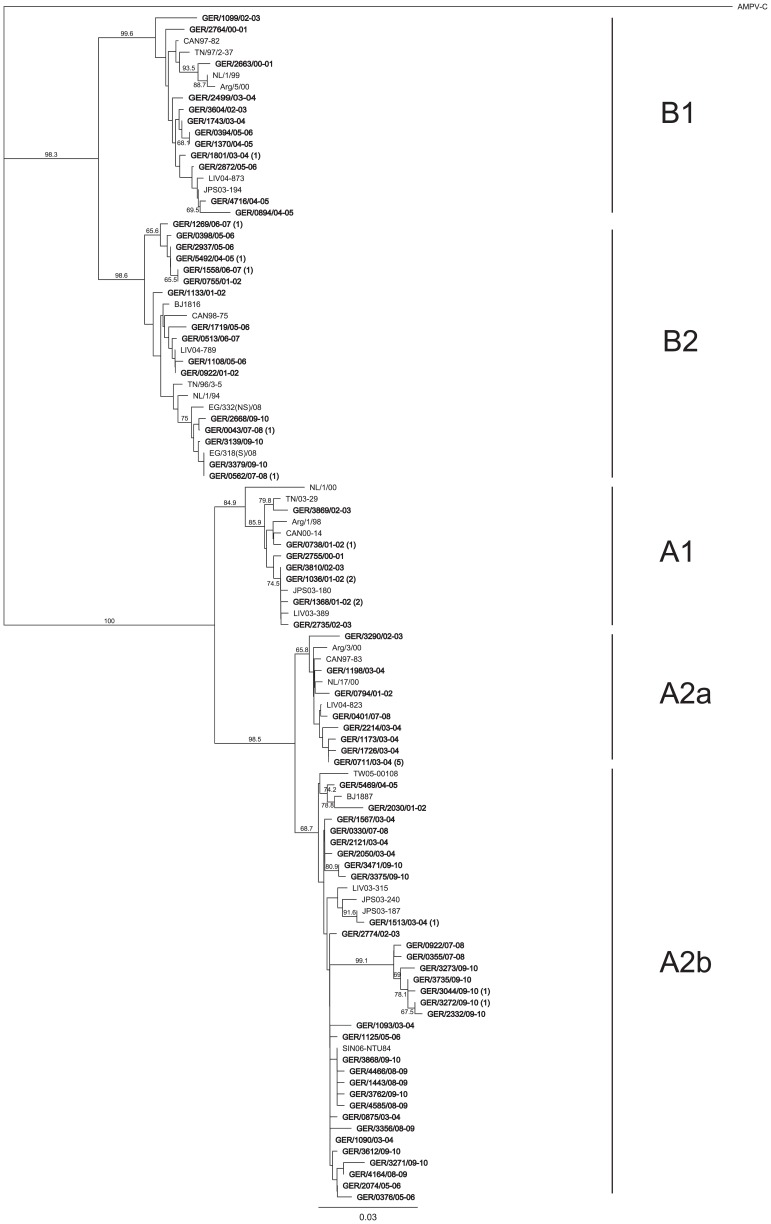
Phylogenetic tree of partial F gene fragments of German HMPV. The tree was constructed using the maximum likelihood estimation with 1,000 replicates through Geneious 5.0.4. Avian metapneumovirus C (AMPV C) was included in the analysis and used as outgroup. Reference sequences (supporting information Table S1) representing the different HMPV genetic lineages were additionally included in the analysis. German HMPV sequences are shown in boldface. Nomenclature for German sequences uses a letter code representing the country of detection (e.g., “GER” represents Germany) followed by the patient number and season in which the virus was detected. In the case of identical sequences only one sequence is shown, whereas the number in brackets indicates the number of additional identical sequences. The lineages and sub-clusters are indicated to the right of the figure. Only bootstrap values greater than 65% are displayed at the branch nodes.

Phylogenetic analysis was conducted on German HMPV sequences of 477 nt of the F gene (Figure 1). During the 10-year study all of the known HMPV lineages and sub-clusters which are A1, A2, B1, B2, A2a and A2b were detected. Most of the sequences belonged to HMPV subgroup A and were predominantly found to be sub-clusters A2b. Furthermore, there were German HMPV sequences which were identical or closely related to sequences from Singapore (SIN06-NTU84), Japan (JPS03-187, JPS03-180, JPS03-194), Beijing/China (BJ1887), The Netherlands (NL/17/00) and Canada (CAN97-83), Tennessee/USA (TN/03–29), Liverpool/UK (LIV03-180, LIV04-873, LIV04-789) or Egypt (EG/332(NS)/08 and EG/318(S)/08). The majority of these sequences were related to sequences of the same season. Some clusters in lineages B1 and B2, as well as in sub-cluster A2b were only presented by German viruses. One cluster is supported by a high bootstrap value of 99.1%. Moreover, multiple identical sequences were identified during the same season [e.g. GER/0738/01-02(1), GER/1036/01-02(2), GER/1368/01-02(2), GER/1801/03-04(1), GER/1513/03-04(1), GER/0711/03-04(5), GER/5492/04-05(1), GER/1558/06-07(1), GER/1269/06-07(1), GER/0562/07-08(1), GER/3868/09–10(1), GER/3044/09-10(1), GER/3272/09-10(1)]. Limited identical sequences were found in consecutive [GER/1036/01-02(2) and GER/3810/02-03, GER/1370/04-05 and GER/0394/05-06, GER/0562/07-08(1) and GER/3379/09-10] or distant seasons [GER/0755/01-02 and GER/1558/06-07(1)].

### Seasonal Circulation Pattern of HMPV Lineages and Sub-clusters

**Figure 2:**
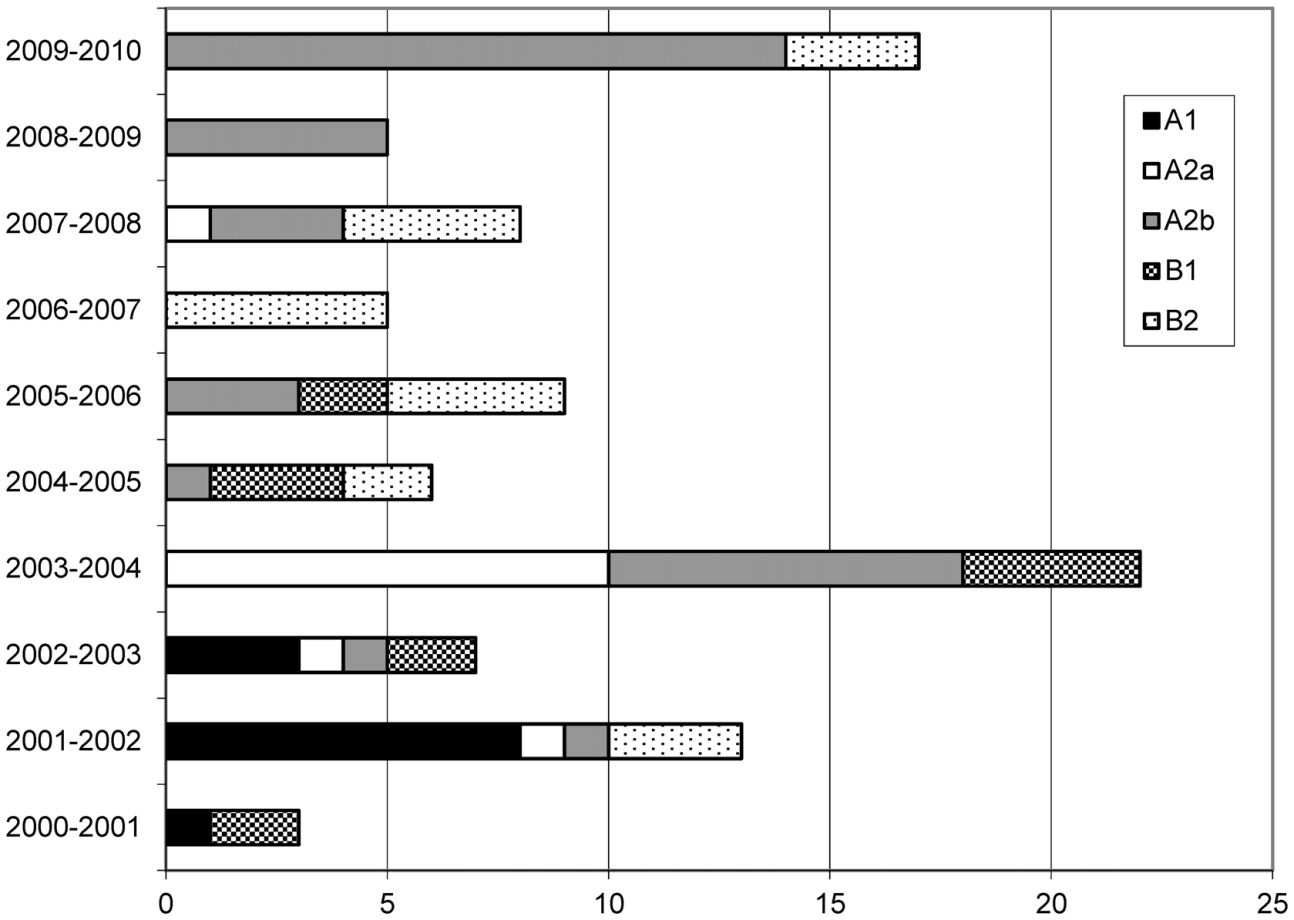
Seasonal circulation of HMPV lineages and sub-clusters in Germany. The number of samples is shown on the abscissa.

With the exception of the seasons 2000–2001 and 2006–2007, all seasons were characterized by the circulation of HMPV A2. Only A2b circulated in all seasons as indicated above (Figure 2). Sub-cluster A2a viruses circulated from 2001–2002 to 2003–2004 and reappeared in the season 2007–2008. Epidemic seasons are further characterized by the recurrent appearance and disappearance of lineage B2, whereas lineage A1 and B1 were only present until seasons 2002–2003 and 2005–2006, respectively.

### Genetic Variability/Diversity of HMPV

Nucleotide distance within genetic lineages and sub-clusters ranged between 0.0056 and 0.0169, whereas the lowest differences were observed for lineage A1 and A2. As expected, nucleotide distance was highest between lineages and sub-clusters where it was in the range of 0.0269–0.1478. Within and between lineages and sub-clusters, nucleotide distances were higher compared to the observed amino acid distances, which were 0.0000–0.0065 within and 0.0000–0.0363 between lineages and sub-clusters, respectively.

Further the translated sequences were compared towards amino acid changes. Sequence comparisons identified the five amino acid residues (positions 233, 286, 296, 312 and 348) specific to each lineage described by Yang et al., 2009 [9]. In addition, single amino acid substitutions were found for the lineages A2 (Asn298Ser) and B1 (Val231Ala, Ser347Thr). The highest amino acid variability was observed for sequences of the lineage B2. Herein, three variations were present: Thr223Asn (7/21 sequences), Asp280Asn (7/21 sequences), and Asn358Lys (1/21 Sequences). Phylogenetic analysis (Figure 1) revealed that these sequences clustered separately and were closely related to sequences from Egypt (EG/332(NS)/08 and EG/318(S)/08).

## Discussion

To study the molecular and epidemiological features of HPMV in Germany a retrospective long-term study was conducted. More than 4,500 clinical specimens of patients with ILI were collected by general practitioners over a period of ten consecutive seasons (2000–2001 to 2009–2010), and HMPV was detected in 138 (3%) respiratory samples. HMPV infections occurred in individuals of all ages but tend to be most frequent in children aged between 0 and 4 years and the elderly (>60 years), which is consistent with previous reports [23]. In a comparable system of national virological surveillance from England and Wales, sentinel general practices detected HMPV in 2.2% of samples collected from patients of all ages with ILI during the winter of 2000–2001 [15]. In contrast to our study, detection of HMPV in northern Greece was on average associated with a twofold higher prevalence (6.05%) in specimens from ILI-patients during the influenza seasons 2005–2006, 2006–2007 and 2007–2008 [24]. But as observed for Germany, in the Greek population HMPV was determined in all age groups, with the highest prevalence in children aged between 0 and 5 years. Studies indicated that HMPV has infected almost all children by the age of 5 years [1], [2]. HMPV infection is not restricted to the very young children but also occurs in adults and elderly subjects [3]. Despite the HMPV infection in childhood, new infections may occur throughout life due to incomplete protective immune responses and/or acquisition of a new lineage [5]. These findings may explain intense frequency in children and the elderly observed in our study.

Symptoms and diagnoses primarily associated with HMPV-positive specimens (ILI) in this study were cough and bronchitis as reported previously [3], [20], [25]. The assessment of odds ratio showed, that in children (<4 years with ILI) cough and bronchitis were associated with HMPV infection. In the age group 5–15 years, HMPV-positive patients had a higher chance to develop pneumonia. Fever/shivers seems to be associated with the elderly (>60 years old) infected with HMPV. However, it was reported that older patients (66–83.2 years old) were less likely to report fever in contrast to adults with an influenza virus infection [26]. Our data support the assumption that clinical manifestations of HMPV infections in adults are not limited to few specific clinical features, but vary from asymptomatic to mild upper RTI symptoms and severe pneumonia, respectively [27]. The comparison of inpatients and outpatients younger than 4 years old revealed that cough is associated most frequently with HMPV-positives of both cohorts, whereas fever/shivers and bronchitis are very common symptoms and diagnoses in HMPV-outpatients. Pneumonia is a frequent diagnosis of hospitalized children positive for HMPV [28]–[30], which did not correlate to our calculated odds ratios. This suggests that, compared to adults, children do not show specific clinical features for a HMPV-associated disease.

Most of the available data on HMPV infections are from studies of young hospitalized children, in which the virus causes acute respiratory illness (ARI) [31]–[34]. HMPV has been detected in 1–17% of ARI cases [23], [35]–[38]. In the present study hospitalized pediatric patients (0–4 year) with ARI were compared to children at the same age with ILI as outpatients. HMPV was detected in 11.9% of specimens from inpatients younger than 4 years in comparison to 6.9% infections in outpatients.

By trend, there was a biennial pattern in seasons with low and high HMPV-positive rates in hospitalized patients from season 2000–2001 to 2006–2007 within the range of 1.4% to 32.3%. Two earlier studies from Germany analysed HMPV in hospitalized children, reporting on similar findings [13], [39]. These data revealed that a season (2001–2002) with a high (17.5%) positive rate was followed by a season (2002–2003) with a low (1.9%) and again by a season (2003–2004) with a high (7.9%) detection rate. In the subsequent seasons the HMPV rate remained in a lower range (6.7% to 10.9), but 2009–2010 there was an increase to 32.8%. Interestingly, the data of HMPV infection rate in outpatients was less distinct than in inpatients. The rates were high in season 2003–2004 (12.0%) and in season 2009–2010 (16.8%). In season 2009–2010 the amount of the positive rate was twice lower in inpatients than in outpatients. Whereas in season 2001–2002 the prevalence of HMPV in inpatients was at a high level with 32.3%, the positive rate in outpatients was at a low level of 2.2%. A reason for this inhomogeneity remains unclear and may be explained by sampling bias or by virulence factors of HMPV. In our study, the sampling bias was reduced by the selection of related amounts of samples over the whole study period versus outpatients and inpatients. Another possible explanation regards the virulence of HMPV. The assumption that HMPV-positive children in the age group of 0–4 years showed severe symptoms and diagnoses and were therefore hospitalized instead of being attend to pediatrician might be an explanation for the high positive rate of inpatients. The HMPV-sequences of the F protein in the season 2001–2002 provide no indication of a cluster with virus variants which may be correlated to changes in the virulence. In addition, we could not find a correlation between a statistically high HMPV prevalent season and a dominance of an antigenic subgroup because in two seasons subgroup A was predominant and in one season it was subgroup B. Seasonal variation of HMPV was observed in other studies with hospitalized patients, like Austria (3–10.1%), Sweden (0.8–5.9%), Italy (7–43%) or South Africa (3.7–9.6%) [23], [40]–[42]. Further studies are necessary to explain the complexity of HMPV frequency rates.

About two thirds of HMPV-positive samples were detected in February and March, indicating that in Germany HMPV circulated primarily during the late winter and early spring, as observed for other countries of the temperate zone [15]–[19]. In Austria, a clear biennial pattern of alternating winter (peak activities between December and February) and spring (peak activities between April and June) activity was observed whereas a significantly higher prevalence of HMPV was determined in seasons with winter activity and *vice versa* [40]. These findings are supported by a study from China in which the authors found HMPV infection peaks between November and February and April to June [28]. HMPV epidemics twice a year were not determined in our study because the sampling was done only during the cold season. With reference to our data, there were only two seasons (2003–2004 and 2009–2010) with a statistically significant intense circulation of HMPV as shown by the population based study. The seasonality of HMPV infection is not fully understood but it can be affected by climate [28] and the timing of seasonal epidemics may vary by region [43].

Phylogenetic analysis of the HMPV F gene of viruses circulating in Germany revealed a complex and dynamic circulation of all HMPV subgroups (A and B) and lineages (A1, A2, B1 and B2) in the ten years study interval. In nine of ten seasons HMPV subgroup A or B predominated the season. In season 2007–2008 both groups were simultaneously existent (Fig. 2). Such a complex pattern was also found in a long-term study from the United States [9]. Predominance of subgroups A or B, respectively, was observed both for a single season (1982, 1983, 1987, 1993, 1994, 2000, 2001, and 2003) and a maximum of three consecutive seasons (1984–1986, 1989–1991, and 1996–1998). Besides, there were seasons with a co-dominance of HMPV subgroups A and B (1988, 1992, 1995, and 1999). Thus, for the United States no regular cyclic pattern could be determined. German HMPV represented all previously described sub-clusters, and co-circulation of mainly two or three sub-clusters was observed. Co-circulation of two or more sub-clusters is not unusual as similar findings have been reported from countries all over the world, e.g. United States [9], Australia [12], [44], United Kingdom [45], Croatia [36], Japan [46], China [47], South Korea [48], Brazil [49] and France [50]. It was suggested, that outbreaks of HMPV subgroups and sub-clusters appear to be a local phenomenon unlike to influenza, which spreads across the continents with two or three viruses [30]. Long-term studies of HMPV for more than two to three seasons have rarely been reported. Only one study in France described the situation of HMPV infections in hospitalized children over a time period of seven years [50]. Therefore it remains difficult to map the European circulation pattern of HMPV. The reason of HMPV circulation complexity remains unclear but it may complicate the challenges for the development of HMPV vaccines.

The analysis of sequence diversity for the F fragment showed that nucleotide distance within lineages/sub-cluster was 0.0056–0.0169. The nucleotide distance was higher than the corresponding amino acid distance (0.0000–0.0065), which was explained by a hypothesis that functional constraints on paramyxovirus fusion proteins prevent dramatic amino acid changes [9]. Within one epidemic season circulation of multiple identical isolates was observed in Germany, implicating that HMPV infections occur due to highly similar viruses within one season. Detection of identical sequences in consecutive seasons is rare. Sequences from German isolates were identical to closely related viruses isolated in distant regions suggesting that human travelling behaviour might influence the evolution of new epidemic viruses in distant places.

The amino acid alignment of all German HMPV F sequences showed five amino acid substitutions at positions 233; 286; 296; 312; and 348 that can be used as signature amino acids to differentiate between HMPV subgroups A and B [9]. Whereas no specific amino acid residue was found to discriminate between sequences from lineages A1 and A2, there was one specific substitution at position 296 distinguishing between lineages B1 (Asn) and B2 (Asp) [9]. Specific amino acid substitutions are not limited to German sequences and were also specific to sequences from the United States [9], The Netherlands [11] or Canada [14]. Sequence analysis of the complete F gene determined a number of amino acid residues distinct to a certain subgroup or lineages, however, the biological importance of these variations is not clear [9]. Moreover, we observed two amino acid substitutions which were specific for seven German HMPV of lineage B2. These viruses cluster separately with sequences from Egypt possessing the same substitutions [51]. Prospectively, it has to be monitored if those viruses develop into a new sub-cluster in lineage B2.

In summary, the present study provides for the first time data on HMPV infections in in- and outpatients of the German population. HMPV was detected in individuals of all ages during ten consecutive seasons, mainly in February and March. HMPV infections were associated with cough, fever/shivers and pneumonia, respectively. Molecular characterization of HMPV revealed a complex circulation pattern of group dominance where HMPV groups A or B viruses predominated in general for three consecutive seasons. German HMPV represented all of the previously described lineages and sub-clusters. Next to the existence of a time-dependent circulation of subgroups and sub-clusters co-circulation of mainly two or three lineages was observed. Furthermore, nucleotide diversity was greater than amino acid diversity within lineages, suggesting that HMPV does not evolve rapidly which is an important feature for the development of monoclonal antibodies and vaccine strains. Our HMPV data present the first long-term study in Germany in in- and outpatients. It is one of only few in Europe and therefore it provides new insights into the epidemiology and genetic diversity of HMPV in Europe and, moreover, allows comparative analyses of worldwide circulating HMPV.

### Limitations of the Study

Like most published studies on HMPV, the present study has limitations. This includes potential sampling biases that may affect accurate risk factor modelling. In this study, the rate of HMPV-positives was in general higher in inpatients than in outpatients. This observation might be explained by the different selection criterion of these groups. Samples from inpatients come from patients with ARI, which is described to be similar to HMPV in children [16], [33], [43]. In contrast, samples from outpatients were taken from patients with ILI. Influenza-like illness is preferentially a clinical manifestation of HMPV in adults but not in children or the elderly [3]. As only outpatients presenting with ILI symptoms were sampled, most acute respiratory infections that occur outside this surveillance definition are unsampled, thereby increasing the likelihood of underestimation HMPV as a cause of ARI in children and the elderly. The query of symptoms and diagnoses was not consistent for bronchitis in ILI patients (outpatients) because the questionnaire was changed during the ten year sampling period in this aspect. Nevertheless, the rate of bronchitis was high and our data may underestimate the overall rate of bronchitis in HMPV-positive outpatients, particularly because no data of bronchitis in outpatients were collected in the season 2003–2004 with an intensive circulation of HMPV.

## Supporting Information

Table S1 **HMPV GenBank sequences^a^.**
(DOCX)
