# Supplementary material for: Human Metapneumovirus: Insights from a Ten-Year Molecular and Epidemiological Analysis in Germany
Source: PLoS One. 2014 Feb 5;9(2):e88342. doi: 10.1371/journal.pone.0088342 (PMC3914980; doi:10.1371/journal.pone.0088342)
Supplement: Table S1 — HMPV GenBank sequencesa. (DOCX) [file pone.0088342.s001.docx]

TABLE S1. HMPV GenBank sequences^a^

| Strain | GenBank accession no. | Country of isolation |
| --- | --- | --- |
| AMPV-C | AY590688 | United States |
| Arg/1/98 | DQ362939 | Argentina |
| Arg/3/00 | DQ362941 | Argentina |
| Arg/5/00 | DQ362945 | Argentina |
| BJ1816 | DQ843658 | China |
| BJ1887 | DQ843659 | China |
| CAN00-14 | AY145299 | Canada |
| CAN97-82 | AY145295 | Canada |
| CAN97-83 | AY145296 | Canada |
| CAN98-75 | AY145289 | Canada |
| EG/318(S)/08 | HQ909765 | Egypt |
| EG/332(NS)/08 | HQ909766 | Egypt |
| JPS03-180 | AY530092 | Japan |
| JPS03-187 | AY530093 | Japan |
| JPS03-194 | AY530094 | Japan |
| JPS03-240 | AY530095 | Japan |
| LIV03-315 | EU179272 | United Kingdom |
| LIV03-389 | EU179277 | United Kingdom |
| LIV04-789 | EU179273 | United Kingdom |
| LIV04-823 | EU179274 | United Kingdom |
| LIV04-873 | EU179275 | United Kingdom |
| NL/1/00 | AF371337 | Netherlands |
| NL/1/94 | AY304362 | Netherlands |
| NL/1/99 | AY304361 | Netherlands |
| NL/17/00 | AY304360 | Netherlands |
| SIN06-NTU84 | EF397621 | Singapore |
| TN/03-29 | EU857609 | United States |
| TN/96/3-5 | EU857584 | United States |
| TN/97/2-37 | EU857570 | United States |
| TW05-00108 | DQ841214 | Taiwan |

^a^ HMPV sequences were retrieved from the GenBank database (http://www.ncbi.nlm.nih.gov/nucleotide/)
